# Inflammatory markers response to citrulline supplementation in patients with non-alcoholic fatty liver disease: a randomized, double blind, placebo-controlled, clinical trial

**DOI:** 10.1186/s13104-019-4130-6

**Published:** 2019-02-15

**Authors:** Zahra Darabi, Mina Darand, Zahra Yari, Mehdi Hedayati, Amirhosein Faghihi, Shahram Agah, Azita Hekmatdoost

**Affiliations:** 1 Department of Clinical Nutrition and Dietetics, Faculty of Nutrition Sciences and Food Technology, National Nutrition and Food Technology Research Institute, Shahid Beheshti University of Medical Sciences, #7, West Arghavan, Farahzadi Blv, Shahrake Gharb, Tehran, Iran; 2 Cellular and Molecular Endocrine Research Center, Research Institute for Endocrine Sciences, Shahid Beheshti University of Medical Sciences, Tehran, Iran; 3 Colorectal Research Center, Iran University of Medical Sciences, Tehran, Iran

**Keywords:** Citrulline, NAFLD, Fatty liver, Inflammation, NASH

## Abstract

**Objectives:**

The aim of this study was to investigate the effects of citrulline (Cit) supplementation on inflammatory markers and liver histopathology in patients with non-alcoholic fatty liver disease (NAFLD). In this clinical trial, fifty NAFLD patients were assigned to receive 2 g/day Cit or placebo for 3 months.

**Results:**

At the end of study, serum high sensitive C-reactive protein (hs-CRP) and activity of nuclear factor kappa B (NF-κB) were reduced in Cit group significantly more than placebo group (P-value = 0.02 and < 0.01 respectively). Serum concentrations of tumor necrosis factor-α (TNF-α) was reduced in Cit group significantly more than placebo after adjusting for levels of baseline (P-value < 0.001). Moreover, Cit supplementation decreased serum alanine aminotransferase (ALT) and hepatic steatosis significantly (P = 0.04). Anthropometric measurements and hepatic enzymes did not change significantly in any group (P ≥ 0.05). In conclusion, our results showed that 12 weeks supplementation with 2 g/day Cit improved inflammatory markers in patients with NAFLD. Further studies with longer period of supplementation and different dosages of Cit are needed to be able to conclude.

*Trial registration* IRCT201703194010N18 on 2017-10-13

## Introduction

Non-alcoholic fatty liver disease (NAFLD) begins from simple steatosis, and if it is not treated it may progress to non-alcoholic steatohepatitis (NASH), cirrhosis, and even hepatocellular [1–3]. Inflammation and lobular ballooning result from insistent hepatic injury then they can cause nonalcoholic steatohepatitis (NASH) and lastly may lead to cirrhosis [1]. NAFLD and NASH are reversible, while cirrhosis exhibit an irreversible stage of the disease [4]. The only proven treatment for NAFLD is lifestyle modification [2, 5], which can be more effective when combined with some dietary supplements [6–10].

Specific amino acids such as Citrulline (Cit) supplementation are suggested as effective strategies in amelioration of hepatic steatosis and insulin resistance (IR) in experimental models of NAFLD. They could improve hepatic steatosis through their effects on glucose tolerance, IR and lipid metabolism [11–14]. Some studies have shown potential effects of Cit on reducing inflammatory markers such as tumor necrosis factor α (TNF-α) and interleukin-6 (IL-6) [12, 15, 16], which can prevent development of disease to severe conditions such as NASH and cirrhosis [17]. The aim of current study was to investigate effects of Cit supplementation on liver enzymes, inflammatory indices, hepatic elasticity, and echogenicity in patients with NAFLD.

## Main text

### Materials and methods

#### Ethical consideration

The study protocol was approved by the National Nutrition and Food Technology Research Institute of Shahid Beheshti University of Medical Science (IR.SBMU.nnftri.13950106). The trial was registered at Iranian registry of clinical trials (IRCT201703194010N18).

#### Study design and participants

The study designed as a prospective randomized, Double-Blind, placebo-controlled, clinical trial. Patients with NAFLD were recruited from a private Hepatology clinic. NAFLD was diagnosed by a gastroenterologist according to Fibroscan results. Inclusion criteria included age older than 18 year and controlled attenuation parameter (CAP) score more than 260 in Fibroscan. Exclusion criteria included pregnancy and lactation in women, viral hepatitis, intake of alcohol, insulin injections and consuming hepatotoxic medications.

After explanation of study protocol, patients who agreed to participate in the study were randomly assigned to receive four capsules/day of either placebo (maltodextrin) or Cit (0.5 g) for 12 weeks. Cit capsules were filled by 500 mg l-citrulline Maleate, provided by Mardin Company, Tehran, Iran. Capsules containing Cit or placebo were identical in shape, size and color. Capsules containing Cit or placebo were placed in boxes, and labeled as A or B by a third person so that investigators and patients were blinded on group assignments. Both groups have been advised on an energy-balanced diet according to Clinical Guidelines on the Identification, Evaluation, and Treatment of Overweight and Obesity in Adults from the National Institutes of Health and the North American Association [18]. Follow-up assessments have been implemented every 4 weeks after start of intervention. Compliance was evaluated through capsule count in each visit.

#### Clinical and paraclinical assessments

Weight, height, and waist circumferences of all participants were measured. BMI was calculated using the following formula: BMI = weight (kg)/height^2^ (m). Serum alanine aminotransferase (ALT) and aspartate aminotransferase (AST) were measured by photometric assay (Parsazmoun). Levels of gamma glutamine transferase (GGT) was measured by enzymatic colorimetric assay (Parsazmoun). Concentrations of fasting TNF-α (TNF-α; BOSTER, Pleasanton, CA, USA) and high-sensitivity C_reactive protein (hs-CRP) were measured by using an enzyme-linked immunosorbent assay (Bionik). Nuclear factor kappa-light-chain-enhancer of activated B cells (NF-κB) p65 was assessed in peripheral blood mononuclear cell (PBMC) nuclear extracts using ELISA kits (Cell Signaling) according to the manufacturer’s protocol. Hepatic steatosis and fibrosis were measured using FibroScan, and Ultrasound exam, which weredone at the beginning and end of the intervention by a hepatologist and a radiologist respectively. Assessment of physical activity was done at baseline and end of intervention by employing the metabolic equivalent of task (MET) questionnaire [19].

#### Dietary assessment

Three 24-h recalls for 2 weekdays and 1 weekend day were asked from participants at the beginning and end of study. Interviews were done by an expert dietitian. Nutrient intakes were assessed using Nutritionist 4 (First DataBank) [20].

#### Statistical analysis

Data analyzed using SPSS21 software. Continuous and categorical data were presented as mean values with their standard deviations and frequency. Analyze of demographic variable were performed using t test or χ^2^, as appropriate. Normal distribution of data was evaluated using the Kolmogorov–Smirnov. Paired t test and student t test were used to compare variables within and between groups respectively. In order to exclude the effects of confounding factors, the analysis of covariance test was used. All ANCOVA models were adjusted for the baseline value of each variable and mean changes in BMI, MET and energy.

The sample size was calculated for the Fibroscan controlled attenuation parameter (CAP) score. Determining sample size for this study was based on detection of a 10 unit difference in the mean CAP score with a power of 80% (β = 20%), yielding a sample size of 21 for each group. Due to the potential loss of samples, 25 patients in each group were considered [9].

### Results

**Fig. 1:**
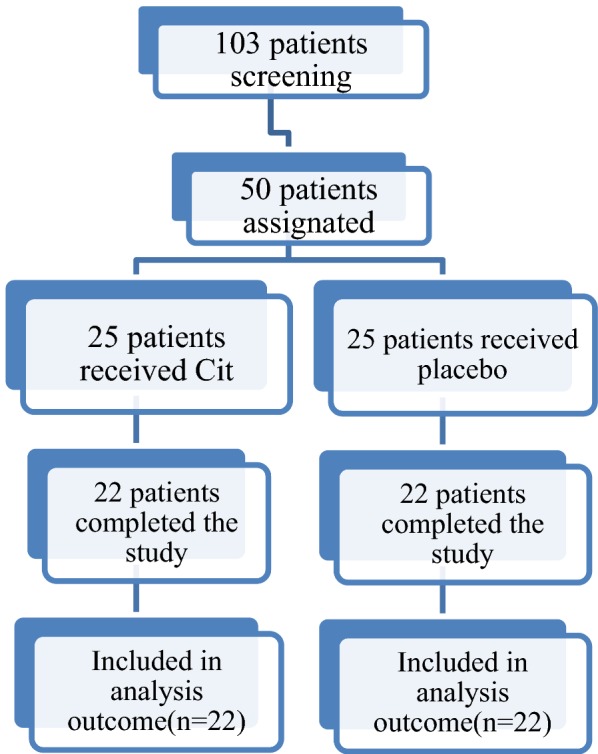
Flowchart of study participants’ enrolment

Fifty patients who met the study inclusion criteria were enrolled in this study. The flow chart of study enrollment is shown in Fig. 1. Eighty-three percent of patients completed 12 weeks of study protocol. Only three (12%) patient in the Cit group and three (12%) patient in the placebo group discontinued the study for personal reasons. The demographic, inflammatory and biochemical characteristics and liver histological parameters of two groups were similar at the baseline, only levels of TNF-α was significantly higher in placebo group at baseline.

There were no significant differences in waist circumference, BMI and weight between and within groups. Serum ALT was significantly reduced in Cit group after 12 weeks intervention period, while serum AST and GGT did not change significantly in any group.

NF-κb activity and hs-CRP reduced significantly in the Cit group compared with the baseline and the placebo group (Table 1). After adjusting for levels of baseline, TNF-α was reduced in Cit group significantly more than placebo group (P = 0.049).

**Table 1:** Inflammatory biomarkers of patients at baseline and after 3 month

Characteristics	Baseline Mean ± SD	After 3 month Mean ± SD	P*	Change Mean ± SD	P^†^
*hs-CRP (ng/ml)*					
Cit	5696 ± 2862	3901 ± 2760	< 0.01	− 1794 ± 2025	0.02
Placebo	6323 ± 4812	6143 ± 4851	0.713	− 180.6 ± 1866	
*TNF-α (pg/ml)*					
Cit	15.1 ± 4.74	14.6 ± 2.01	0.66	− 0.55 ± 0.07	0.57
Placebo	18.5 ± 2.04	17.4 ± 1.57	0.05	− 1.29 ± 2.37	
*NF-κb (ng/mg protein)*					
Cit	2.46 ± 0.72	2.11 ± 0.42	0.02	− 0.30 ± 0.63	< 0.001
Placebo	1.99 ± 1.34	2.64 ± 0.72	0.09	0.64 ± 1.29	

*TNF-α* tumor necrosis factor α, *hs-CRP* C_reactive protein, *NF-κB* nuclear factor kappa B cells

* P values indicate comparison within groups

^†^P values indicate comparison between the changes of each variable between two groups

At the end of the study, results of Fibroscan showed a significant reduction of Hepatic steatosis and fibrosis in the Cit group; however, this reduction was not significantly different between groups (P > 0.05). According to Ultrasound exam, hepatic steatosis grade reduced in Cit group significantly more than placebo group (Table 2).

**Table 2:** Liver steatosis and fibrosis of patients at baseline and after 3 month (Mean values and standard deviations)

Characteristics	Baseline Mean ± SD	After 3 month Mean ± SD	P*	Change Mean ± SD	P^†^
*Fibrosis (kPa)*					
Cit	5.85 ± 1.71	5.84 ± 2.00	0.44	− 0.21 ± 1.18	0.8
Placebo	6.27 ± 2.08	6.12 ± 1.81	0.15	− 0.43 ± 1.15	
*Steatosis grade (0/1/2/3)* ^*‡*^					
Cit	2.86 ± 0.35	2.44 ± 0.85	0.04	− 0.44 ± 0.85	0.8
Placebo	2.62 ± 0.66	2.38 ± 0.88	0.13	− 0.37 ± 0.95	
*Steatosis (dB/m)*					
Cit	320.8 ± 29.6	304.5 ± 39.1	0.127	− 16.33 ± 43.18	0.97
Placebo	315.5 ± 36.8	299.6 ± 45.7	0.171	− 15.87 ± 44.19	

* P values indicate comparison within groups

^†^P values indicate comparison between the changes of each variable between two groups

^‡^According to ultrasound exam

### Discussion

To our knowledge, this study is the first randomized, clinical trial that evaluated the effect of Cit supplementation on markers of inflammation in patient with NAFLD. Results of this study have shown that consumption of 2 g/day Cit reduced markers of inflammation significantly.

The results of previous experimental studies are consistent with our results. Experimental studies have shown that Cit supplementation can reduce gene expression of Toll-like receptor 4 (TLR4), which results in inhibition of NF-kB activation and TNF-α production [11, 15, 16].

Another suggested mechanism for anti-inflammatory effects of Cit is its properties in reduction of oxidative stress. Cai et al. reported that Cit supplementation increased superoxide dismutase (SOD) activities and reduced levels of malondialdehyde (MDA) [21]. SOD can reduce extracellular signal-regulated protein kinases 1 and 2 (ERK1/2) signaling activation. ERK1/2 inhibition leads to prevention of NF-kB activation and TNF-α production [22].

Moreover, Sellmann et al. showed that Cit supplementation protects animals fed a high fructose diet against hepatic lipid peroxidation. Increased lipid peroxidation is the main cause of redox sensitive nuclear factor κB activity and consequently production of TNF-α [23, 24].

Our results showed reduction in serum ALT, and hepatic steatosis in the Cit group; however there was no significant difference between two groups. Results of two experimental studies have shown that Cit supplementation along with high-fat diet or 60% fructose diet can prevent the induction of raising hepatic steatosis in rat [11, 13]. This discrepancy might be due to low dosage of our supplements and/or short duration of the study.

Previous studies reported that Cit down regulated genes expression of Sterol regulatory element-binding transcription factor 1 (SREBF1) and carbohydrate-responsive element-binding protein (CHREBP), fatty acid synthesis (Fas) that decreased de novo lipogenesis (DNL). Cit reduced gene expression SREBF1 by affecting on mammalian target of rapamycin (mTOR) pathway and protein kinase B [13, 25, 26]. All these mechanism are involved in development of hepatic steatosis. Thus, another possibility for difference in our results with these studies is that prevention and reduction of hepatic steatosis may require different mechanisms. Though Cit supplementation was able to prevent fatty liver, its activity in treating fatty liver might be not as clear as that in prevention, particularly when administered at low dosages and short duration.

The important advantage of this study is that it is the first clinical trial that evaluated the effects of Cit on inflammation markers in human patients with NAFLD. Other strengths of this study is evaluating NF-κB activity in PBMC. Furthermore, participants were newly diagnosed patients with NAFLD; so, they did not receive any treatment before and during the study.

### Conclusion

In conclusion, our results showed that 12 weeks supplementation with 2 g/day Cit improved inflammatory markers in patients with NAFLD. Further studies with longer period of supplementation and different dosages of Cit are needed to be able to conclude.

## Limitations

One of the limitations of this study was that we did not use different dosages of Cit. We could not use higher dosages of Cit because we did not find enough evidence for their safety in human. Secondly, we could not obtain liver biopsy to estimate hepatic steatosis, and fibrosis. Liver biopsy is gold standard for the evaluation of liver fibrosis during of chronic liver diseases. However transient elastography (FibroScan^®^) used that provides quantitative, safely evaluation of NAFLD by scaling hepatic steatosis (CAP score) and fibrosis. This technique was a reliable, noninvasive way for identification of patients with significant hepatic steatosis and fibrosis. It is readily, replicability and its score has low inter- and intra-observer variability [27, 28]. Thirdly, the clinical significance of reduced inflammatory markers is not clear so additional studies are needed to see if these reductions translate to less liver- or cardiovascular-related endpoints.

## Abbreviations

Cit: citrulline
NAFLD: none-alcoholic fatty liver disease
ALT: alanine aminotransferase
AST: aspartate aminotransferase
GGT: γ-glutamyl transferase
IR: insulin resistance
NASH: none-alcoholic steatohepatitis
TNF: tumor necrosis factor
NF-kB: nuclear factor kappa B
hs-CRP: high sensitive C reactive protein
BMI: body mass index
SREBF1: sterol regulatory element-binding transcription factor 1
mTOR: mammalian target of rapamycin
Fas: fatty acid synthesis
DNL: de novo lipogenesis
CREBP: carbohydrate-responsive element-binding protein
